# First Mesozoic entomofauna from the Qinghai-Tibetan Plateau

**DOI:** 10.1016/j.fmre.2026.04.013

**Published:** 2026-04-17

**Authors:** Qianqi Zhang, Jun Chen, Jiahao Li, Chunpeng Xu, Zhengyu Song, Yan Fang, Daran Zheng, Edmund A. Jarzembowski, Haichun Zhang, Bo Wang

**Affiliations:** a College of Paleontology, Shenyang Normal University, Shenyang 110034, China; b State Key Laboratory of Palaeobiology and Stratigraphy, Nanjing Institute of Geology and Palaeontology, Chinese Academy of Sciences, Nanjing 210008, China; c Key Laboratory of Evolution of Past Life in Northeast Asia, Ministry of Natural Resources, Shenyang 110034, China; d Institute of Geology and Paleontology, Linyi University, Shuangling Road, Linyi 276000, China; e University of the Chinese Academy of Sciences, Beijing 100049, China; f Institut für Zoologie und Evolutionsforschung, Friedrich-Schiller-Universität Jena, 07743 Jena, Germany; g School of GeoSciences, Yangtze University, Wuhan 430100, China; h Department of Earth Sciences, The Natural History Museum, London SW7 5BD, United Kingdom; i University of Chinese Academy of Sciences, Nanjing 211135, China

The Qinghai-Tibetan Plateau, as the highest plateau on Earth and ‘the Third Pole’, has witnessed the origin and evolution of the Tibetan biotas [1]. Mesozoic non-marine sediments are widely distributed in the northern (Qaidam Basin) and eastern parts of the Qinghai-Tibetan Plateau, containing abundant terrestrial fossils (plants and animals) [2]. Despite being the most diverse terrestrial flying animals on Earth, only a few Mesozoic insects have been reported from this plateau [3]. Here, we report a diverse fossil insect assemblage from the Lower Jurassic Xiaomeigou Formation in the Qaidam Basin, which represents the first known Mesozoic entomofauna from the Qinghai-Tibetan Plateau.

We collected 118 fossil insects from the Hongshankuangou outcrop (37°51ʹ59.39ʺ N, 95°15ʹ54.77ʺ E; H = 3141 m) in Dachaidan Town, Haixi Mongolian and Tibetan Autonomous Prefecture, Qinghai Province (Fig. S1a-b online) and therefore name them as the Dachaidan Entomofauna. The insect-bearing Lower Jurassic Xiaomeigou Formation is composed mainly of coarse fluvio-lacustrine sandstone and mudstone. The fossils were obtained from the grey mudstone intervals with interbedded coal layers in the upper member of this formation (Fig. S1c–e online). The majority of fossil insects (about 82%) are complete, suggesting they underwent only short-distance transport before burial. The insect fragments consist entirely of allochthonous terrestrial forms, primarily represented by beetle elytra and cockroach wings.

The Dachaidan Entomofauna is characterized by a diverse array of species, predominantly comprising Coleoptera (beetles; 48%), followed by Blattodea (cockroaches; 26%), Plecoptera (stoneflies; 12%), and Hemiptera (bugs; 6%). As the most abundant order, beetles are dominated by archostematans and polyphagans. In particular, some specimens display intricate morphological features in exquisite detail (Fig. 1c, e and f). Some can be attributed to the genus *Zygadenia* Handlirsch, 1906 (*Zygadenia dachaidanensis* Song et al., 2025, *Z. haixiensis* Song et al., 2025), which is widely distributed in the Jurassic and Lower Cretaceous of Asia [3]. The Blattodea are the second most common order of the entomofauna, and the cockroaches are attributed to families Caloblattinidae Vršanský & Ansorge, 2000 and Liberiblattinidae Vršanský, 2002 (Figs. 1b, d; S2b–d online), all of which are extinct families of cosmopolitan Mesozoic cockroaches. Their wings exhibit a high mutation load (Figs. 1d; S2b–d online), suggesting a potential ecological stress in this assemblage [4]. Plecoptera are also abundant, with adults and nymphs. These adults can be assigned to the extinct family Perlariopseidae Sinitshenkova, 1985 (Fig. 1a). Perlariopseidae is a keystone insect group for stratigraphical correlation and paleoecological comparison because it was widely distributed in the Jurassic and Cretaceous of Asia [5]. Most fossils of this family are isolated wings. The well-preserved adults from Dachaidan therefore have important implications for understanding the morphology and evolution of Perlariopseidae. In addition, some well-preserved specimens belong to plant-sucking hemipterans (Fig. S2g online). The presence of both terrestrial insects (beetles, cockroaches, and hemipterans) and aquatic stoneflies indicates that the Dachaidan insects lived in a swamp environment, supported by the coal-bearing fluvial sequences [6].

**Fig. 1:**
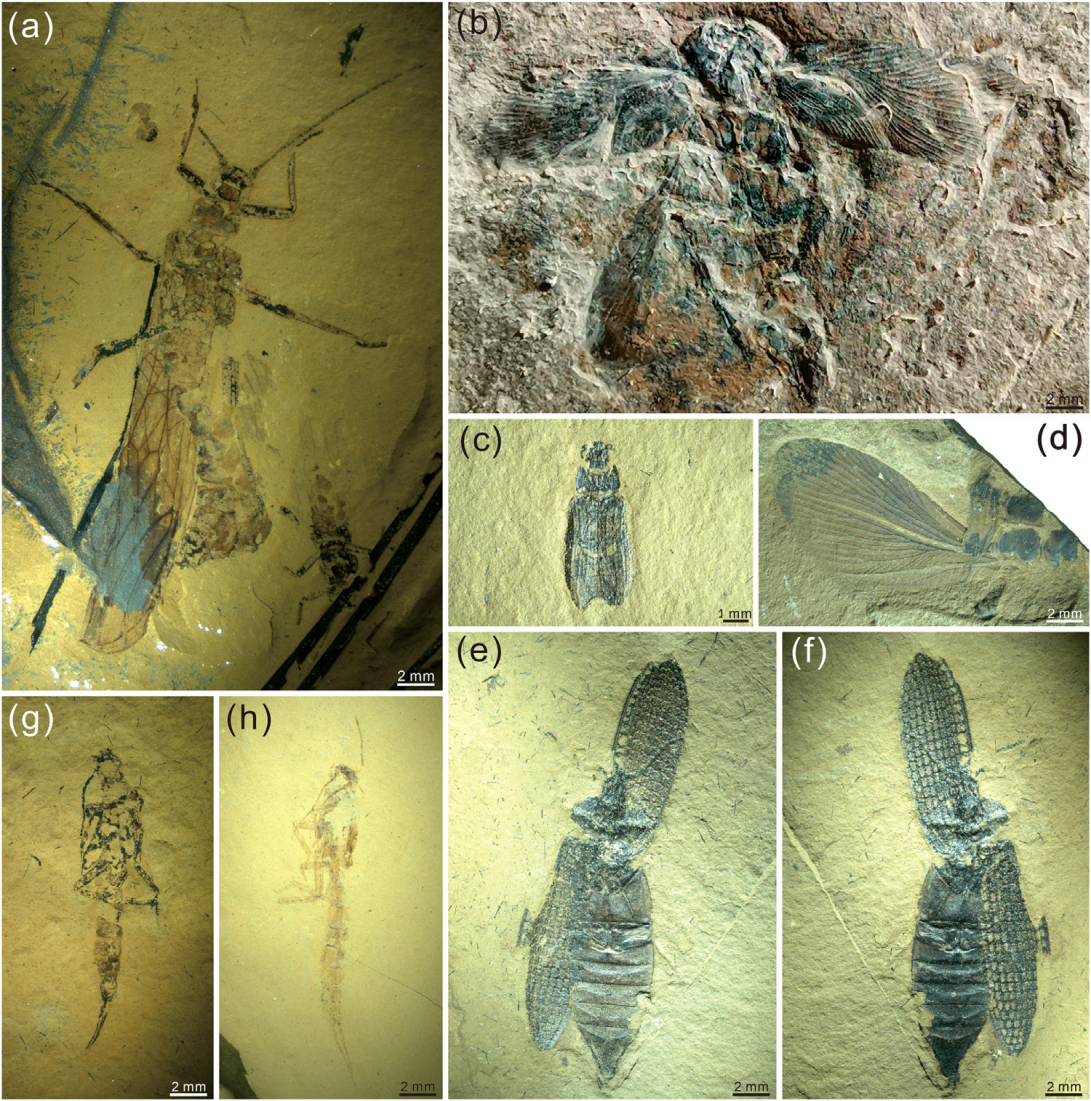
**Representative fossils from Dachaidan Entomofauna.** (a) stonefly (Plecoptera: Perlariopseidae Sinitshenkova, 1985); NIGP210168; (b) cockroach (Blattodea: Caloblattinidae Vršanský & Ansorge, 2000), NIGP210169; (c) beetle (Coleoptera: *Zygadenia* Handlirsch, 1906), NIGP205538; (d) cockroach (Blattodea: Caloblattinidae Vršanský & Ansorge, 2000), NIGP210170; (e, f) beetle (Coleoptera: *Zygadenia* Handlirsch, 1906), NIGP205539a, b; (g, h) stonefly nymphs (Plecoptera), NIGP210171 and NIGP210172. Scale bars = 1 mm for (c), 2 mm for others; (c). (a, g and h) imaged with alcohol wetting, others dry.

In China, two significant Early Jurassic insect entomofaunas were previously reported. The Xiwan Entomofauna in northeastern Guangxi is dominated by mayfly swarms (Ephemeroptera) preserved in yellow mudstone and was buried in a low kinetic energy lacustrine environment [7]. The entomofauna in Karamay, dominated by terrestrial scorpionflies (Mecoptera) and froghoppers (Hemiptera), was entombed in a boggy, peaty and volcanic depositional environment [8]. In striking contrast to these two entomofaunas, the Dachaidan Entomofauna is a mixture of aquatic insects and allochthonous terrestrial insects. In particular, the Dachaidan aquatic insect assemblage belongs to the Type B lacustrine assemblage based on the abundant stoneflies and sedimentological features such as coal-bearing fluvial sequences [9]. This assemblage type was characterized by a detritivore-based aquatic food web, in which primary production consisted of coarse and fine detritus and dead benthic algae [10]. This assemblage inhabited shallow oxbow lakes in large river valleys and represents a hypotrophic ecosystem that is characterized by a suite of physical, chemical, and biological features that were distinct from other contemporaneous and later ecosystems [10]. This extinct hypotrophic ecosystem was previously thought to be restricted to the Early Jurassic-Early Cretaceous of southern Siberia, western Mongolia and northern Kazakhstan (Fig. S3 online) [9]. Our findings expand the paleogeographical distribution of this unique ecosystem to northern China. This result further highlights the significant differences among Early Jurassic entomofaunas on different tectonic plates in China.

The Cenozoic fossil insects of the Qinghai-Tibetan Plateau were sporadically reported during the past 4 decades, such as in the Paleocene Niubao Formation (Qiangtang Basin), Oligocene Dingqing Formation (Lunpola and Dingqing basins) and lower Miocene Garang Formation (Gonghe-Guide Basin). Although abundant Mesozoic plants have been reported from the Qinghai-Tibetan Plateau [2], Mesozoic insects were poorly known previously. The Dachaidan Entomofauna represents the first and only record of Mesozoic insect assemblages from the Qinghai-Tibetan Plateau, and also the first record of a hypotrophic ecosystem in China. It not only holds the potential for a deeper understanding of the evolution and paleogeographical distribution of Jurassic entomofaunas, but also offers a unique opportunity for the reconstruction of a Jurassic terrestrial ecosystem on the Qinghai-Tibetan Plateau. More importantly, this finding highlights the potential to reveal more fossil insect localities with further exploration in the Second Tibetan Plateau Scientific Expedition.

## CRediT authorship contribution statement

**Qianqi Zhang:** Writing – original draft, Funding acquisition. **Jun Chen:** Investigation, Funding acquisition. **Jiahao Li:** Investigation. **Chunpeng Xu:** Investigation. **Zhengyu Song:** Investigation. **Yan Fang:** Investigation. **Daran Zheng:** Investigation. **Edmund A. Jarzembowski:** Writing – review & editing. **Haichun Zhang:** Writing – review & editing, Investigation. **Bo Wang:** Writing – review & editing, Writing – original draft, Methodology, Investigation, Funding acquisition, Conceptualization.

## Declaration of competing interest

The authors declare that they have no conflicts of interest in this work.

## Appendix Supplementary materials

Image, application 1

## References

[1] Chen F.H., Ding L., Piao S.L. (2021). The Tibetan Plateau as the engine for Asian environmental change: The Tibetan Plateau Earth system research into a new era. Sci. Bull..

[2] Liu X.Y., Yang X.J., Zhou Z.Y. (2007).

[3] Song Z.Y., Jarzembowski E.A., Xiao C.T. (2025). New Jurassic notocupedins (Coleoptera: Archostemata: Ommatidae) from the Qinghai-Xizang (Tibetan) Plateau. Palaeoworld.

[4] Vršanský P., Oružinský R., Aristov D. (2017). Temporary deleterious mass mutations relate to originations of cockroach families. Biologia.

[5] Liu Y.S., Sinitshenkova N.D., Ren D. (2009). A revision of the Jurassic stonefly genera *Dobbertiniopteryx* Ansorge and *Karanemoura* Sinitshenkova (Insecta: Plecoptera), with the description of new species from the Daohugou locality, China. Paleontol. J..

[6] Huang X.H., Sun Y.Q., Wang W.C. (2020). Sequence-palaeogeography and coal accumulation from the early and middle Jurassic in the Xidatan Area of the Northern Qaidam Basin. Acta Sedimentol. Sin..

[7] Zhang Q.Q., Wang B., Zheng D.R. (2022). Mayflies as resource pulses in Jurassic lacustrine ecosystems. Geology.

[8] Zheng D.R., Wang H., Nel A. (2019). A new damsel-dragonfly (Odonata: Anisozygoptera: Campterophlebiidae) from the earliest Jurassic of the Junggar Basin, northwestern China. Alcheringa.

[9] Sinichenkova N.D., Zherikhin V.V. (1996). Mesozoic lacustrine biota: Extinction and persistence of communities. Paleontol. J..

[10] L.A. Buatois, C.C. Labandeira, M.G. Mángano, et al., Chapter 11 The Mesozoic Lacustrine Revolution, in: M.G. Mángano, L.A. Buatois (Eds.), The Trace-Fossil Record of Major Evolutionary Events, Topics in Geobiology 40. Dordrecht, 2016, pp. 179-263.

