# Supplementary material for: First Mesozoic entomofauna from the Qinghai-Tibetan Plateau
Source: Fundam Res. 2026 Apr 17;6(4):2366–8. doi: 10.1016/j.fmre.2026.04.013 (PMC13424976; doi:10.1016/j.fmre.2026.04.013)
Supplement: Supplementary file 1 [file mmc1.docx]

Supplementary Materials:

Geological setting, Material and methods

Figures: S1-S3

References: 4

Submitted to *Fundamental Research*

**Geological Setting**

The Hongshankuangou section lies across the limbs of a synclinal structure, not far north of the National Highway 315 (Fig. S1b online). Jurassic strata exposed in the study area are (top downwards): the Xiaomeigou Formation (Figs. S1c-d online) (J_1_: coal-bearing beds), Dameigou Fm. (J_2_: coal-bearing beds), Shimengou Fm. (J_2_: fine sandstones) and Caishiling Fm (J_2_: coarse sandstones). (Huang et al., 2020). They were mainly deposited in Mesozoic-Cenozoic fan delta, shallow-moderately deep lake, fluvial, swamp and delta facies (Wang, 2012). The coal-bearing Xiaomeigou Formation is a fossiliferous deposit within which many palaeobotanical taxa have been recorded (Li et al., 1988).

**Material and Methods**

All the fossil specimens were collected from the middle part of the Lower Jurassic Xiaomeigou Formation. They were photographed dry or under alcohol using a stereomicroscope system (ZEISS Stereo Discovery V16). Plates were made up with the software CorelDRAW 2019 and Photoshop CC. Fossil preparation, observation and identification were carried out at the Nanjing Institute of Geology and Palaeontology, Chinese Academy of Sciences (NIGPAS) where the specimens are housed.


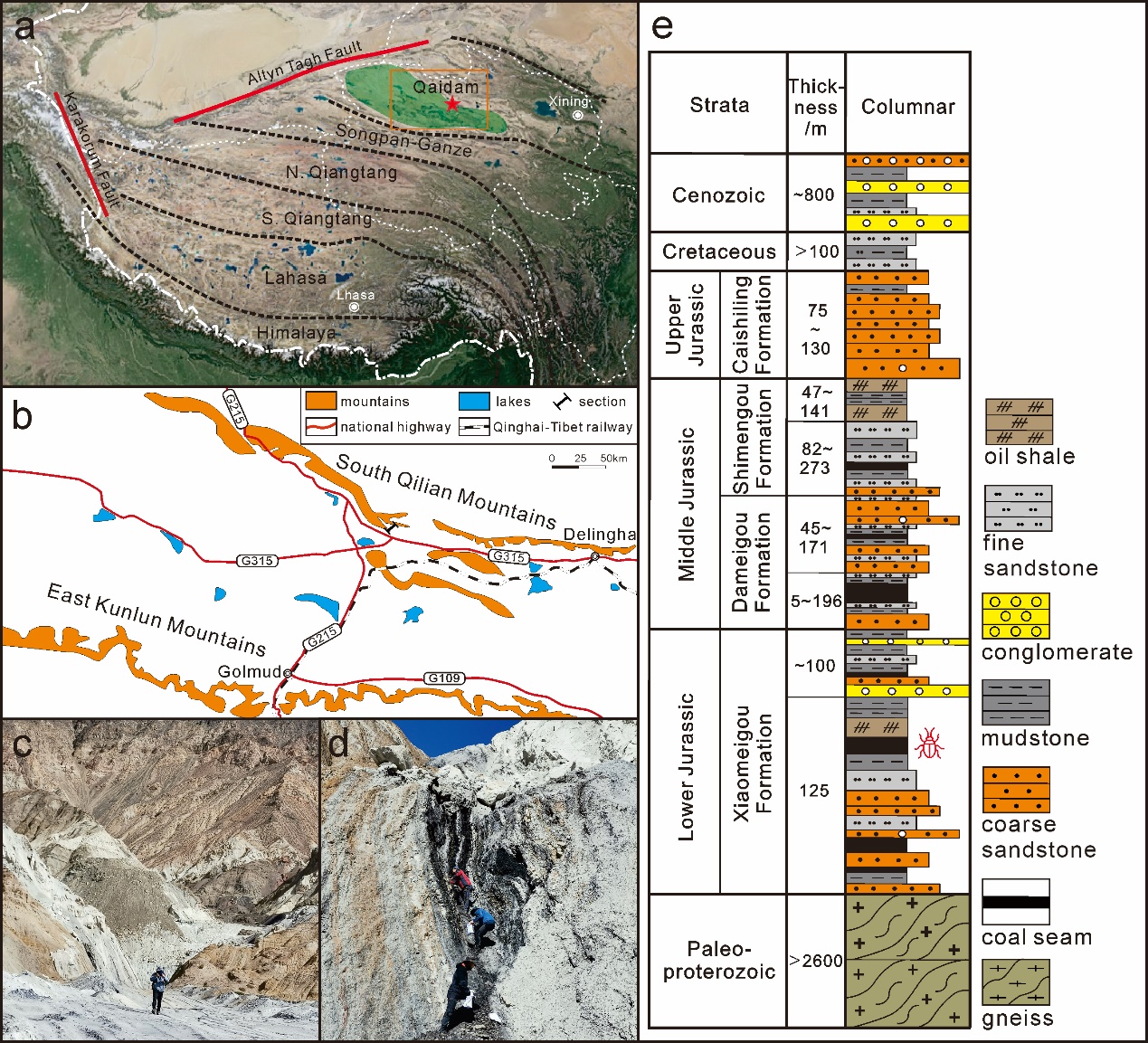


**Fig. S1** (a) Region of the Qaidam Basin (green colour), red star showing study site; (b) General geography and roads in yellow rectangle in (A); (c, d) Outcrop at the Hongshankuangou section; (e) Lithostratigraphic column of the section with sampling horizon (modified from Huang et al., 2020).


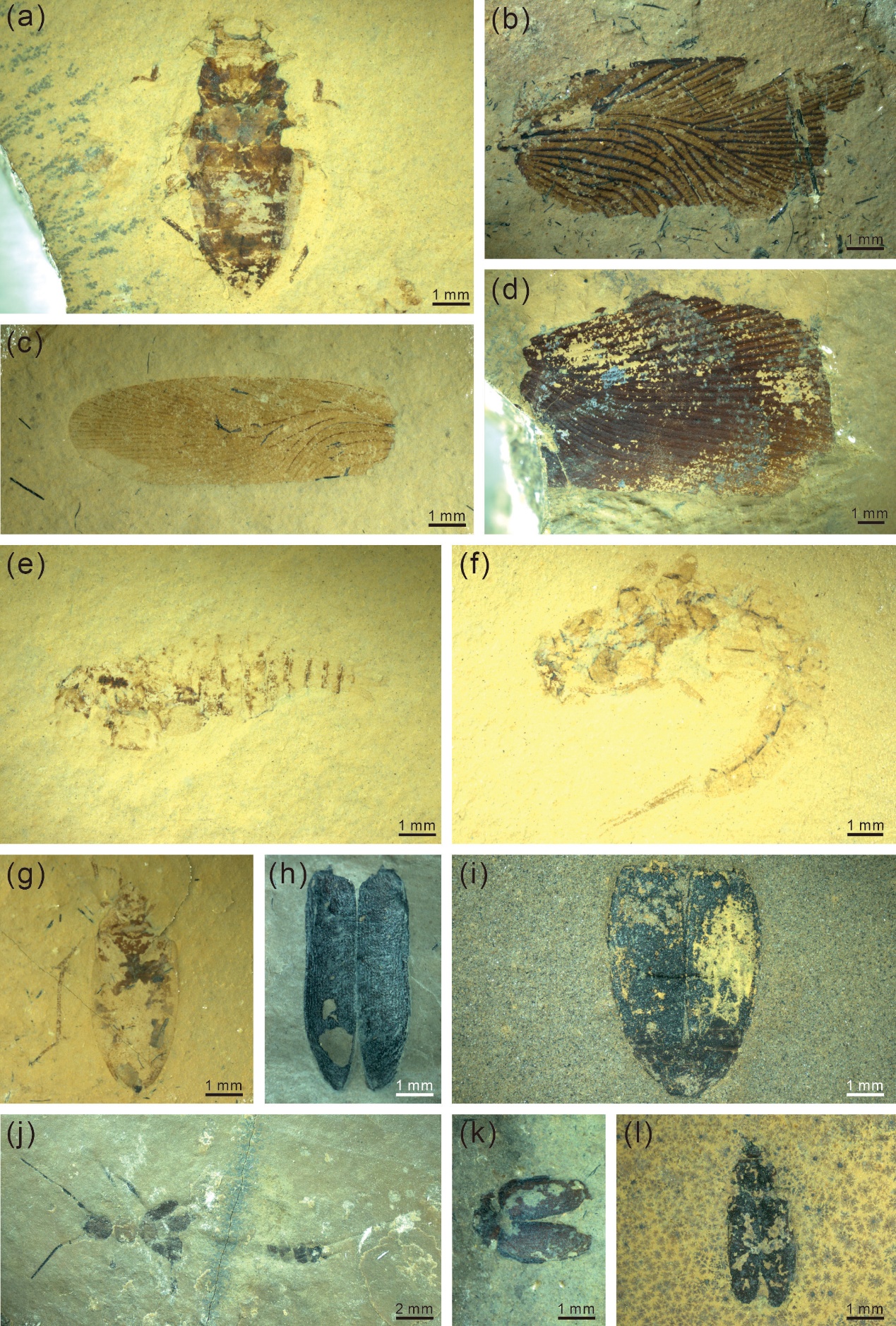


**Fig. S2** Additional representative fossils from the Dachaidan Entomofauna:

(a) beetle (Coleoptera), NIGP210173;

(b) cockroach (Blattodea: Caloblattinidae: *Rhipidoblatta* [Vishnyakova, 1968](https://paleobiodb.org/classic/app/refs?#display=8446)), NIGP210174;

(c) cockroach (Blattodea: Liberiblattinidae: *Hra* Vršanský, 2020), NIGP210175;

(d) cockroach (Blattodea: Caloblattinidae: *Rhipidoblatta* [Vishnyakova, 1968](https://paleobiodb.org/classic/app/refs?#display=8446)), NIGP210176;

(e-f) stonefly nymphs (Plecoptera), NIGP210177 and NIGP210178;

(g) bug (Hemiptera), NIGP210179;

(h) beetle (Coleoptera), NIGP210180;

(i) beetle (Coleoptera), NIGP210181;

(j) stonefly nymph (Plecoptera), NIGP210182;

(k) beetle (Coleoptera), NIGP210183;

(l) beetle (Coleoptera), NIGP210184;

Scale bar = 1 mm for all figures.


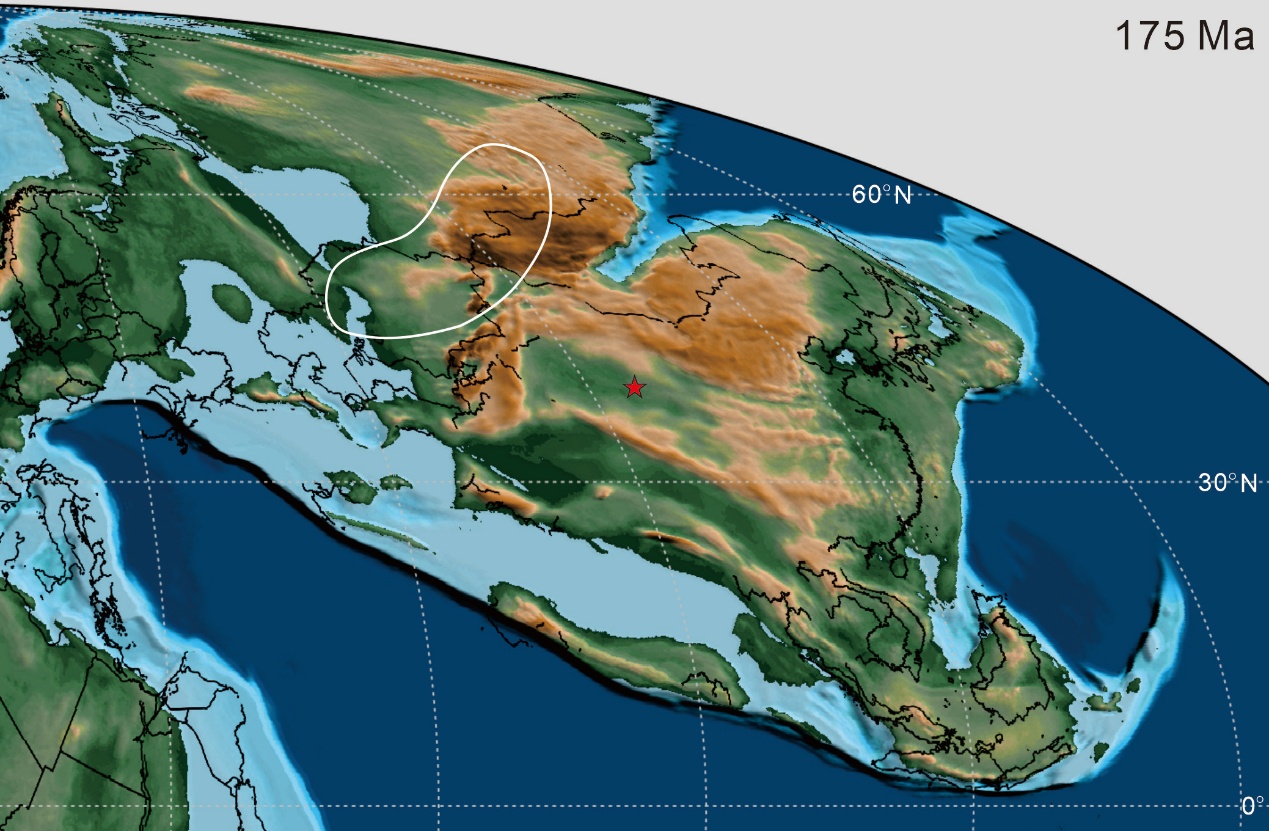


**Fig. S3** Palaeogeographic distribution of Mesozoic hypotrophic ecosystems. Early Jurassic palaeogeographic map is modified from Scotese (2021). The area indicated by the solid line shows the previously known distribution including southern Siberia, western Mongolia, and northern Kazakhstan. The red star indicates the location of the Dachaidan biota.

**References**

[1] X.H. Huang, Y.Q. Sun, W.C. Wang, et al., Sequence-palaeogeography and coal accumulation from the Early and Middle Jurassic in the Xidatan area of the northern Qaidam Basin. Acta Sedimentol. Sin. 38 (2020) 266-283.

[2] P.L. Wang, D.W. Lv, H.Y. Liu, et al., Migration law of Mesozoic Qaidam Basin depocenters. Adv. Mater. Res. 524 (2012) 63-66.

[3] P.J. Li, Y.L. He, X.W. Wu, et al., Early and Middle Jurassic strata and their floras from northeastern border of Qaidam Basin, Qinghai. Nanjing University Press, Nanjing 1988.

[4] C.R. Scotese, An atlas of Phanerozoic paleogeographic maps: The seas come in and the seas go out. Annu. Rev. Earth and Planet. Sci. 49 (2021) 679-728.
